# Impaired muscle morphology in a *Drosophila* model of myosin storage myopathy was supressed by overexpression of an E3 ubiquitin ligase

**DOI:** 10.1242/dmm.047886

**Published:** 2020-12-29

**Authors:** Martin Dahl-Halvarsson, Montse Olive, Malgorzata Pokrzywa, Michaela Norum, Katarina Ejeskär, Homa Tajsharghi

**Affiliations:** 1 Department of Pathology, Institute of Biomedicine, University of Gothenburg, 41345 Gothenburg, Sweden; 2 Institute of Neuropathology, Department of Pathology and Neuromuscular Unit, Department of Neurology, IDIBELL-Hospital de Bellvitge, 08907 Hospitalet de Llobregat, Barcelona, Spain; 3 Translational Medicine, School of Health Sciences, University of Skövde, SE-541 28, Skövde, Sweden

**Keywords:** Slow/β-cardiac myosin heavy chain, *MYH7*, Myosin storage myopathy, *Drosophila* model, Ubiquitin proteasome system, E3 ubiquitin ligase, Potential therapeutic approach

## Abstract

Myosin is vital for body movement and heart contractility. Mutations in *MYH7*, encoding slow/β-cardiac myosin heavy chain, are an important cause of hypertrophic and dilated cardiomyopathy, as well as skeletal muscle disease. A dominant missense mutation (R1845W) in *MYH7* has been reported in several unrelated cases of myosin storage myopathy. We have developed a *Drosophila* model for a myosin storage myopathy in order to investigate the dose-dependent mechanisms underlying the pathological roles of the R1845W mutation. This study shows that a higher expression level of the mutated allele is concomitant with severe impairment of muscle function and progressively disrupted muscle morphology. The impaired muscle morphology associated with the mutant allele was suppressed by expression of Thin (herein referred to as Abba), an E3 ubiquitin ligase. This *Drosophila* model recapitulates pathological features seen in myopathy patients with the R1845W mutation and severe ultrastructural abnormalities, including extensive loss of thick filaments with selective A-band loss, and preservation of I-band and Z-disks were observed in indirect flight muscles of flies with exclusive expression of mutant myosin. Furthermore, the impaired muscle morphology associated with the mutant allele was suppressed by expression of Abba. These findings suggest that modification of the ubiquitin proteasome system may be beneficial in myosin storage myopathy by reducing the impact of *MYH7* mutation in patients.

## Abstract (precis)

**Summary:**

Exploration of the phenotypes associated with the MYH7 R1845W mutation in skeletal muscles, and an alleviating mechanism of the pathological phenotype, suggesting E3-ligase modifier gene activity potentially affects the impact of the mutation.

## INTRODUCTION

Myosin heavy chain (MyHC) is the molecular motor of muscle and forms the backbone of the sarcomeric thick filaments. It converts chemical energy of ATP hydrolysis into mechanical force, which is vital for body movement and heart contractility. Hereditary myosin myopathies have emerged as an important group of muscle diseases with variable clinical and morphological expression, depending on the mutated isoform and location of the mutation (Tajsharghi and Oldfors, 2013). Mutations in slow/β-cardiac MyHC (*MYH7*), expressed in type 1 skeletal muscle fibres and in the heart ventricles (Smerdu et al., 1994), are associated with skeletal and/or cardiac myopathies. Mutations mainly located within the globular head of slow/β-cardiac MyHC are an important cause of hypertrophic and dilated cardiomyopathy (Walsh et al., 2010), whereas mutations located at the α-helical coiled-coil C-terminal rod domain (LMM) cause two skeletal myosin myopathies, Laing distal myopathy and myosin storage myopathy (MSM), with or without cardiac involvement (Tajsharghi and Oldfors, 2013).

Mutations in the LMM region can affect the ability of the protein to form stable and functional thick filaments, based on the amino acid change, the position in the heptad repeat motif (a-b-c-d-e-f-g) (McLachlan and Karn, 1982) and the location in the LMM. MSM is a protein aggregate myopathy associated with myosin accumulation (Tajsharghi et al., 2003). It is caused by primarily dominant mutations located within or close to the 29-residue assembly competence domain in the distal end of the LMM of slow/β-cardiac MyHC (Tajsharghi and Oldfors, 2013), which is known to be critical for the proper assembly of sarcomeric myosin rod filaments (Sohn et al., 1997). Consequently, mutations in this region may cause defective integration of dimers into the thick filament, leading to an accumulation of unassembled MyHC. In contrast, mutations associated with Laing distal myopathy that are situated far from the assembly competence domain might cause other effects on the thick filament structure and function leading to a different pathology (Tajsharghi and Oldfors, 2013). MSM and Laing distal myopathy show distinct morphological and clinical phenotypes, depending on the mutated residue at the tail region (Tajsharghi and Oldfors, 2013).

A dominant missense mutation that changes the highly conserved arginine at position 1845 to tryptophan (R1845W) was the first *MYH7* mutation identified in MSM (Tajsharghi et al., 2003). The mutated residue is located in the outer f position, in which the side chains are available to interact with other myosin dimers or other proteins. This mutation has been reported in several unrelated cases, confirming the association of the R1845W mutation of *MYH7* with MSM (Tajsharghi et al., 2003; Laing et al., 2005; Kiphuth et al., 2010; Pegoraro et al., 2007; Shingde et al., 2006). Muscle biopsy in affected individuals demonstrates characteristic subsarcolemmal accumulation of material restricted to type 1 muscle fibres. The stored material displays myofibrillar ATPase activity and intense immunoreactivity to slow/β cardiac MyHC, thus the term myosin storage myopathy was introduced (Tajsharghi et al., 2003).

The clinical manifestations in MSM patients are intra- and extra-familial highly variable, ranging from mild muscle weakness to severe impairment of ambulation (Tajsharghi et al., 2003; Laing et al., 2005; Pegoraro et al., 2007; Shingde et al., 2006; Stalpers et al., 2011; Cancilla et al., 1971; Ceuterick et al., 1993; Barohn et al., 1994; Bohlega et al., 2003; Masuzugawa et al., 1997; Uro-Coste et al., 2009; Sahgal and Sahgal, 1977). Delayed motor milestones, difficulties in climbing stairs or running, a waddling gait and usually proximal muscle weakness in four limbs have been described in many cases with MSM. The onset of the disease is usually in childhood and the course is mostly slowly progressive and scoliosis sometimes supervenes (Stalpers et al., 2011; Bohlega et al., 2003).

The suitability of *Drosophila melanogaster* as a model organism for myosin myopathies has been demonstrated in previous studies (Viswanathan et al., 2017; Dahl-Halvarsson et al., 2018; Wang et al., 2012; Suggs et al., 2017). This suitability is based on the largely conserved sarcomeric structure of myofibrils between flies and human (Reedy and Beall, 1993), as well as the existence of a single *Mhc* gene producing all Mhc isoforms through alternative RNA splicing (Bernstein et al., 1983). Very recently, we used CRISPR/Cas9 genome engineering to develop the first fly model for Laing distal myopathy to investigate the pathobiological mechanisms of the recurrent K1729del *MYH7* mutation (Dahl-Halvarsson et al., 2018). We showed that the corresponding *Drosophila Mhc^K1728del^* mutant phenotype recapitulated certain muscle morphological phenotypes manifest in Laing distal myopathy patients carrying the K1729del *MYH7* mutation. Furthermore, we identified a potential therapeutic approach involving the ubiquitin proteasome system (UPS), which completely rescued the mutant phenotypes.

In this study, we have combined CRISPR/Cas9 genome engineering and the *UAS-GAL4*-based gene expression system to develop fly models for MSM associated with the recurrent R1845W *MYH7* mutation. The *UAS-GAL4*-based gene expression system was used to investigate the dose-dependent effect and pathobiological mechanisms of the *MYH7* mutation, and to assess underlying mechanisms of the aberrant accumulations of myosin in the muscle fibres of affected individuals with MSM. Moreover, we identified that increased expression of the *Drosophila* protein Thin (herein referred to as Abba), which has essential roles in maintaining sarcomeric integrity (Domsch et al., 2013; LaBeau-DiMenna et al., 2012), alleviates the muscle pathological phenotype.

## RESULTS

### Viability and lifespan of adult flies

The elongated α-helical coiled-coil C-terminal rod domain of MyHC exhibits filament-forming properties that assemble into thick filaments of the sarcomeres. Mutations at the outer f position of the heptad repeats have been predicted to cause improper filament formation through disturbed interaction with other myosin dimers and, thereby, perturb thick filament assembly (Tajsharghi and Oldfors, 2013). We generated fly models for the recurrent R1845W mutation, located at the f position of the heptad repeat of α-helical coiled-coil LMM of *MYH7*, to investigate the underlying mechanisms of the pathological features observed in the muscle fibres of affected individuals with MSM.

**Fig. 1.**
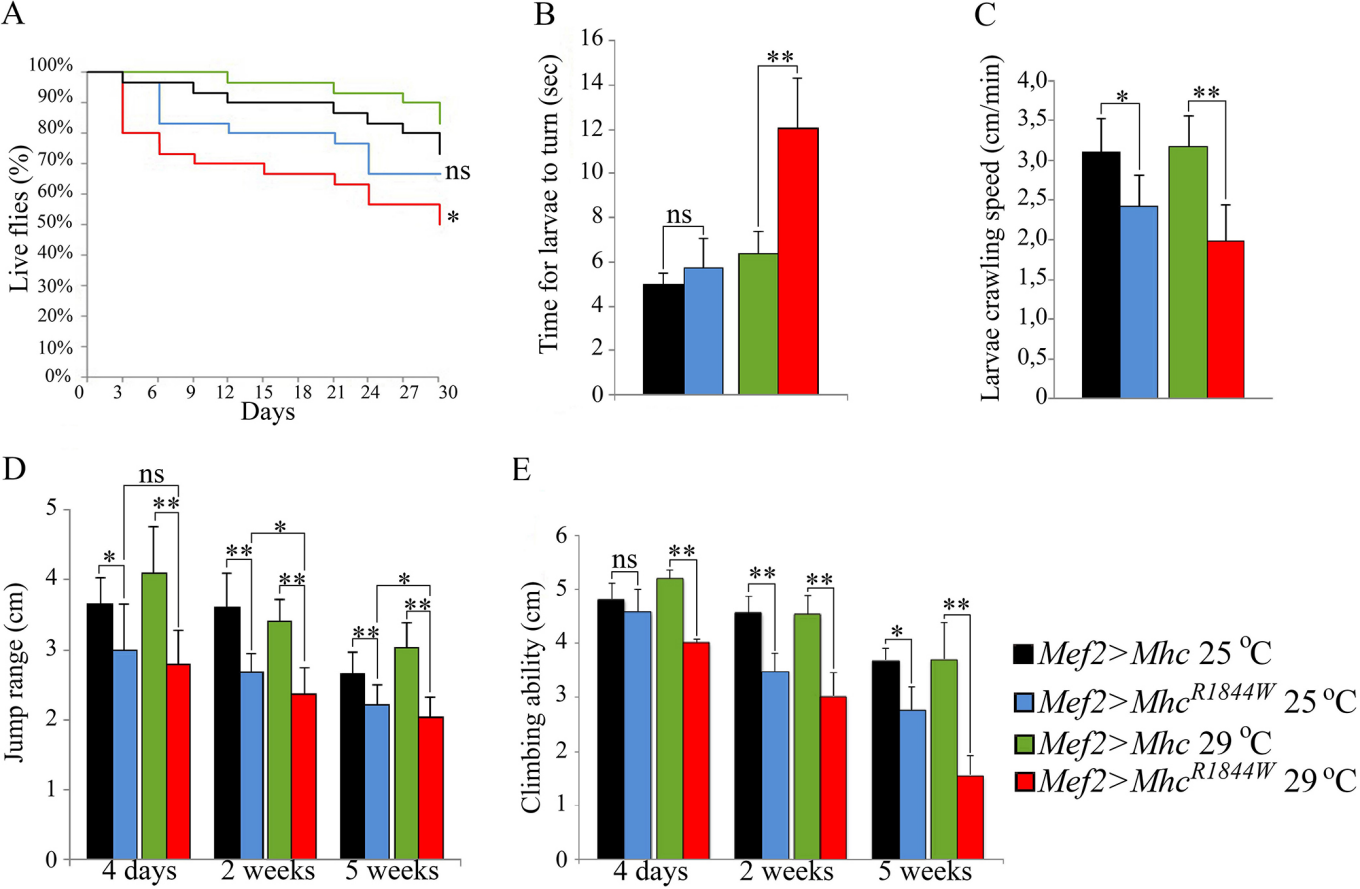
**Impaired muscle function in *eMhc^R1844W^* overexpressing flies.** The phenotypic effect of the e*Mhc^R1844W^* allele was studied by overexpression of either mutated or wild-type e*Mhc* in flies with a heterozygote null *Mhc* allele (*Mhc^1^*) background. (A) *Mef2>eMhc^R1844W^/^+^* adult flies exhibited significantly shorter lifespans compared to control flies (*Mef2>eMhc*) at 29°C (*Mef2>eMhc^R1844W^* versus *Mef2>eMhc*). At 25°C, the mutants had a tendency to have a shorter lifespan but this difference was not significant. (B) At 29°C, the *Mef2>eMhc^R1844W^* larvae showed a significant increase in the time needed to turn belly-down when placed on their backs, whereas no significant increase in time was seen for the *Mef2>eMhc^R1844W^* larvae at 25°C, when compared to controls (*Mef2>eMhc*). (C) *Mef2>eMhc^R1844W^* larvae showed a significant reduction in crawling ability at 25°C, whereas the crawling speed of mutant larvae was reduced to a highly significant degree at the third instar larval stage at 29°C. (D) Jump ability of *Mef2>eMhc^R1844W^* flies are reduced at 25°C and 29°C, compared to controls. At 29°C, *Mef2>eMhc^R1844W^* display a highly significant reduction at all time points. At 25°C, the reduction of jump ability for 4-day-old flies was significant and highly significant for 2-week- and 5-week-old flies. There was no significant difference between 4-day-old *Mef2>eMhc^R1844W^* flies when kept at 25°C and 29°C, but the jump ability of *Mef2>eMhc^R1844W^* flies significantly declined at 2 weeks of age at 29°C, compared to the mutant flies at 25°C. (E) Climbing ability of *Mef2>eMhc^R1844W^* flies at 29°C was severely impaired compared to control flies (*P*<0.001) at all time points measured. There was no significant difference in climbing ability between 4-day-old flies of both genotypes at 25°C, but the deterioration was significant at 2 weeks and 5 weeks of age between *Mef2>eMhc^R1844W^* and control flies. Data are mean±s.e.m. Statistical significance was determined using the Kaplan–Meier estimator. *P<0.05; **P<0.001; ns, not significant.

Despite extensive efforts, we were unable to establish a fly carrying the mutant *Mhc^R1844W^* allele by CRISPR/Cas9-mediated genome editing, suggesting that the severe impact of this mutation is incompatible with viability. Thus, we used the *UAS-GAL4*-based gene expression system to investigate the dose-dependent effect and pathobiological mechanisms of the *MYH7* mutation. Among the different *Mhc* isoforms, we chose the embryonic isoform as it has been shown to support viability (Wells et al., 1996); although in a homozygous condition, embryonic Mhc (eMhc) could not support flight and full jump ability. Yet, the fibres assembled normally and myofibril ultrastructure was not dramatically affected. We found that overexpression of both *eMhc* and *eMhc^R1844W^* using the *Mef2-GAL4* driver was viable when reared at 25°C, and there was no difference in lifespan between the two (Fig. 1A). However, unlike *Mef2>eMhc*, *Mef2>eMhc^R1844W^* flies displayed defects in wing posture similar to those reported for *Mhc^10^* mutants that selectively lack Mhc protein in fast-twitch indirect flight muscles (IFMs) and jump muscles (O'Donnell et al., 1989; Nongthomba et al., 2003). At 29°C, when *Gal4*-mediated overexpression is enhanced, *Mef2>eMhc^R1844W^* animals displayed a significantly shorter mean lifespan when compared with control animals (*Mef2>eMhc*) (*P*<0.05) (Fig. 1A), and a majority had wing posture defects. Flies with exclusive expression of *eMhc^R1844W^*, but not *eMhc*, in indirect flight and jump muscles (*Mef2>eMhc^R1844W^/Mhc^10^*) showed reduced viability, precluding investigation of muscle function and fitness. A majority of these flies showed wing posture defects. Thus, the *eMhc^R1844W^* allele has an impact on animal fitness.

### Reduced motility in animals overexpressing Mef2>eMhc^R1844W^

Larval turning and crawling assays were used to evaluate muscle function in *Mef2>eMhc^R1844W^* larvae at 25°C and 29°C. In larval turning assays at 29°C, mutant *Mef2>eMhc^R1844W^* animals showed a twofold increase in the time needed to turn over and resume crawling on the ventral side compared with control animals with overexpression of wild-type *eMhc* (*Mef2>eMhc*) (*P*<0.001 versus controls) (Fig. 1B). Mutant *Mef2>eMhc^R1844W^* animals showed no significant changes in larval turning assays at 25°C compared with control animals (Fig. 1B). Furthermore, *Mef2>eMhc^R1844W^* larvae exhibited a significant reduction in crawling ability compared to the control animals at 29°C (*Mef2>eMhc^R1844W^*: 2.0 cm versus *Mef2>eMhc*: 3.2 cm, *P*<0.001) (Fig. 1C). There was also a significant reduction in crawling ability between *Mef2>eMhc^R1844W^* and *Mef2>eMhc* animals at 25°C (*Mef2>eMhc^R1844W^*: 2.4 cm versus *Mef2>eMhc*: 3.1 cm, *P*<0.05) (Fig. 1C).

Jump, climbing and flight ability was subsequently analysed in 4-day-old, and 2- and 5-week-old flies with overexpression of either wild-type *eMhc* or mutant *eMhc^R1844W^* allele at 25°C and 29°C. Jump ability of *Mef2>eMhc^R1844W^* flies was impaired at both temperatures. At 29°C, the jump ability of *Mef2>eMhc^R1844W^* flies decreased by 30% compared with control flies *Mef2>eMhc* (*P*<0.001) at all time points measured (Fig. 1D). There was also a significant difference in jump ability between 4-day-old *Mef2>eMhc^R1844W^* and 2- and 5-week-old mutant flies (*P*<0.05). At 25°C, reduced jump ability of *Mef2>eMhc^R1844W^* flies ranged from 20% to 30% between 4-day-old flies and 2- and 5-week-old flies (4-day-old *Mef2>eMhc^R1844W^*: 2.99 cm versus *Mef2>eMhc*: 3.65 cm, *P*<0.05; 2- and 5-week-old *Mef2>eMhc^R1844W^*: 2.67 cm and 2.22 cm versus *Mef2>eMhc*: 3.61 cm and 2.66 cm, *P*<0.001) (Fig. 1D). There was no significant reduction in the jump ability of 4-day-old flies with overexpression of *eMhc^R1844W^* at 25°C compared with *Mef2>eMhc^R1844W^* flies at 29°C. However, the jump ability of 2- and 5-week-old flies with overexpression of *eMhc^R1844W^* at 29°C was significantly reduced compared with *Mef2>eMhc^R1844W^* flies at 25°C (*P*<0.05) (Fig. 1D).

Rapid iterative negative geotaxis (RING) assessment of *Mef2>eMhc^R1844W^* indicated severely impaired climbing ability in 2-week-old flies compared to controls (*P*<0.05; Fig. 1E). Unlike jump ability, there was a significant decrease in climbing ability between 4-day- and 2-week-old flies in both *Mef2>eMhc^R1844W^* and control flies (*P*<0.05; Fig. 1E). *Mef2-Gal4*-driven expression of *eMhc^R1844W^*, but not of *eMhc*, also caused loss of flight ability. Although control flies (*Mef2>eMhc*) were able to leave the open vial within 20 min, *Mef2>eMhc^R1844W^* flies remained in the vial and failed to beat their wings, indicating a complete lack of flight ability.

### Muscle morphology is disrupted upon expression of Mhc^R1844W^ and worsens over time

**Fig. 2.**
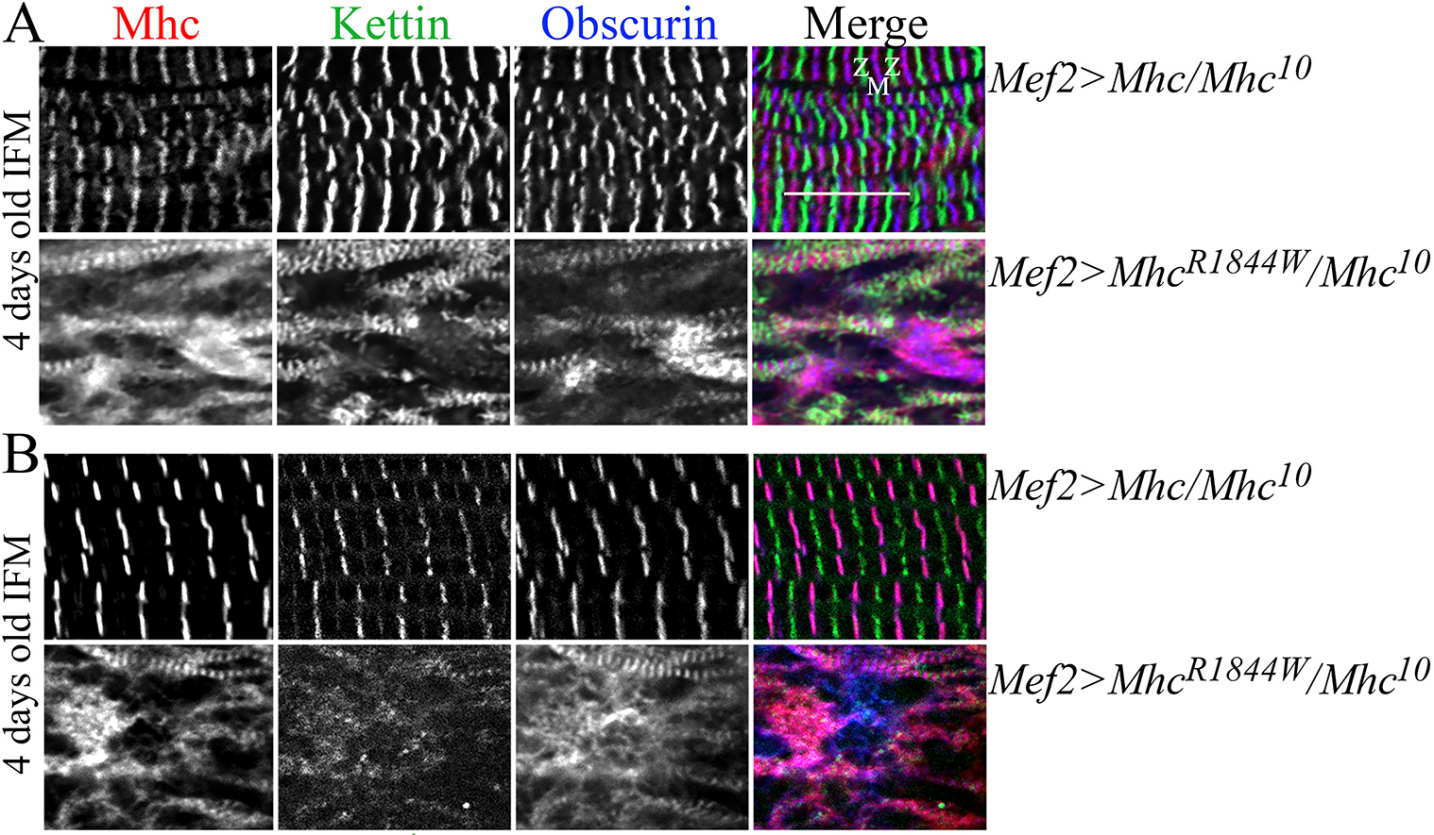
**Muscle morphology in IFMs of 4-day-old *eMhc^R1844W^/Mhc^10^* adult flies.** (A,B) IFMs were labelled for Myosin (Mhc), Kettin/Titin and Obscurin in 4-day-old *Mef2>eMhc* or *Mef2>eMhc^R1844W^* flies carrying the amorphic *Mhc^10^* allele at 25°C (A) and 29°C (B). Each panel shows *Mef2>eMhc* control animals (*w^−^;Mhc^1^,UAS-eMhc/Mhc^10^;Mef2-Gal4/^+^*) (top) and *Mef2>eMhc^R1844W^* animals (*w^−^;Mhc^1^,UAS-eMhc^R1844W^/Mhc^10^;Mef2-Gal4/^+^*) (bottom)*.* In A and B, control flies showed parallel periodic striations across the IFMs. In A, IFMs of the *Mef2>eMhc^R1844W^/Mhc^10^* flies, with exclusive expression of the *eMhc^R1844W^* allele, displayed severe sarcomeric irregularities with few intact sarcomere structures. In B, IFMs of *Mef2>eMhc^R1844W^/Mhc^10^* displayed an absence of periodic sarcomere structure, with some areas showing increased myosin immunofluorescence, probably indicating myosin accumulation. Z and M indicate Z-disks and M-bands, respectively. Scale bar: 10 μm.

The effect of the *Mhc^R1844W^* allele can be addressed in flight and jump muscles by overexpressing mutated or wild-type e*Mhc* in flies carrying the amorphic *Mhc^10^* allele (*Mhc^1^,UAS-eMhc^R1844W^/Mhc^10^;Mef2-Gal4/^+^* and *Mhc^1^,UAS-eMhc/Mhc^10^;Mef2-Gal4/^+^*), at 25°C and 29°C. The *Mhc^1^/Mhc^10^* flies lack functional Mhc in indirect flight and jump muscles. Immunofluorescence analysis of myosin (Mhc), Sallimus (also known as Kettin or Titin), which localises to the Z-disks, and Obscurin (also known as Unc-89) (Katzemich et al., 2012), which links myosin filaments at the M-band of the sarcomere in IFM of four-day-old adult flies, could not detect any clear structural difference between wild-type flies and flies expressing *eMhc* in IFM. Additionally, no differences in muscle structure were detected between animals reared at 25°C and 29°C (Fig. 2A,B), suggesting that an excess amount of *eMhc* in itself might not disturb sarcomere structure. In contrast, and in agreement with a previous study (Viswanathan et al., 2017), we found that IFMs and jump muscles exclusively expressing *eMhc^R1844W^* (*Mef2>eMhc^R1844W^/Mhc^10^*) display severe sarcomeric irregularities in 4-day-old adults at both temperatures. Small areas showed increased myosin immunofluorescence. These undefined subcellular structures were accompanied by more extensive areas of sarcomeric disruption, as evident from mislocalization of Mhc, Sallimus (Kettin) and Obscurin (Fig. 2A,B).

**Fig. 3.**
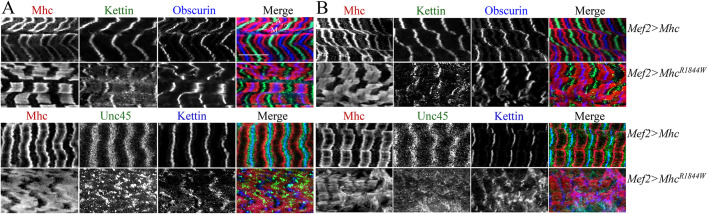
**Muscle morphology in *eMhc^R1844W^* mutants.** (A,B) Larval body wall muscles were labelled for Myosin (Mhc), Kettin/Titin and Obscurin or Mhc, Kettin/Titin and Unc45 at 25°C (A) and 29°C (B). Each panel shows *Mef2>eMhc* control animals (*w^−^*;*Mhc^1^,UAS-eMhc/^+^;Mef2-Gal4/^+^*) (top) and *Mef2>Mhc^R1844W^* animals (*w^−^*;*Mhc^1^,UAS-eMhc^R1844W^/^+^;Mef2-Gal4/^+^*) (bottom)*.* In A, at 25°C, *Mef2>eMhc^R1844W^* larvae displayed disrupted sarcomeric structure, including less defined and thickened A-bands, jagged Z-disks and M-bands. Unc45-containg Z-disks were incompletely defined. In B, at 29°C, at *Mef2>eMhc^R1844W^* animals showed severely disrupted sarcomeric structure and areas with accumulations of Mhc without distinct A-band and wider and jagged Z-disks and M-bands. Unc45 was dispersed throughout the muscle with no clear pattern. Z, A and M indicate Z-disks, A-bands and M-bands, respectively. Scale bar: 10 µm.

To address the effects of *eMhc^R1844W^* expression in animals that also carry a wild-type copy of *Mhc*, we further analysed sarcomere organization in larval body wall muscles from third instar larvae from animals heterozygous for the *Mhc^1^* allele (*w^−^*;*Mhc^1^,UAS-eMhc^R1844W^/^+^;Mef2-Gal4/^+^* or *w^−^*;*Mhc^1^,UAS-eMhc/^+^;Mef2-Gal4/^+^*) and reared at either 25°C or 29°C. *Mhc^1^/^+^;Mef2>eMhc^R1844W^* larvae displayed a severely disrupted sarcomeric structure, including thickened and less defined Mhc-containing A-bands and slightly wider and jagged Z-disks and M-bands in the body wall muscle compared with *Mhc^1^/^+^;Mef2>eMhc* larvae at 25°C (Fig. 3A). The sarcomeric disruption was more severe in animals raised at 29°C, and an increased myosin immunofluorescence was evident in some areas (Fig. 3B). Z-disks and M-bands appeared wider and jagged, and Mhc failed to concentrate in distinct A-bands at either side of the Z-disk and instead occupied nearly the entire space between the Z-disks in *Mef2>eMhc^R1844W^* larvae at 29°C. Immunofluorescence analysis of Unc-45 (which is essential for the folding of Mhc and localises to the Z-disks) *Mef2>eMhc^R1844W^* larvae displayed undefined Unc-45-containing Z-disks at both temperatures (Fig. 3A,B).

**Fig. 4.**
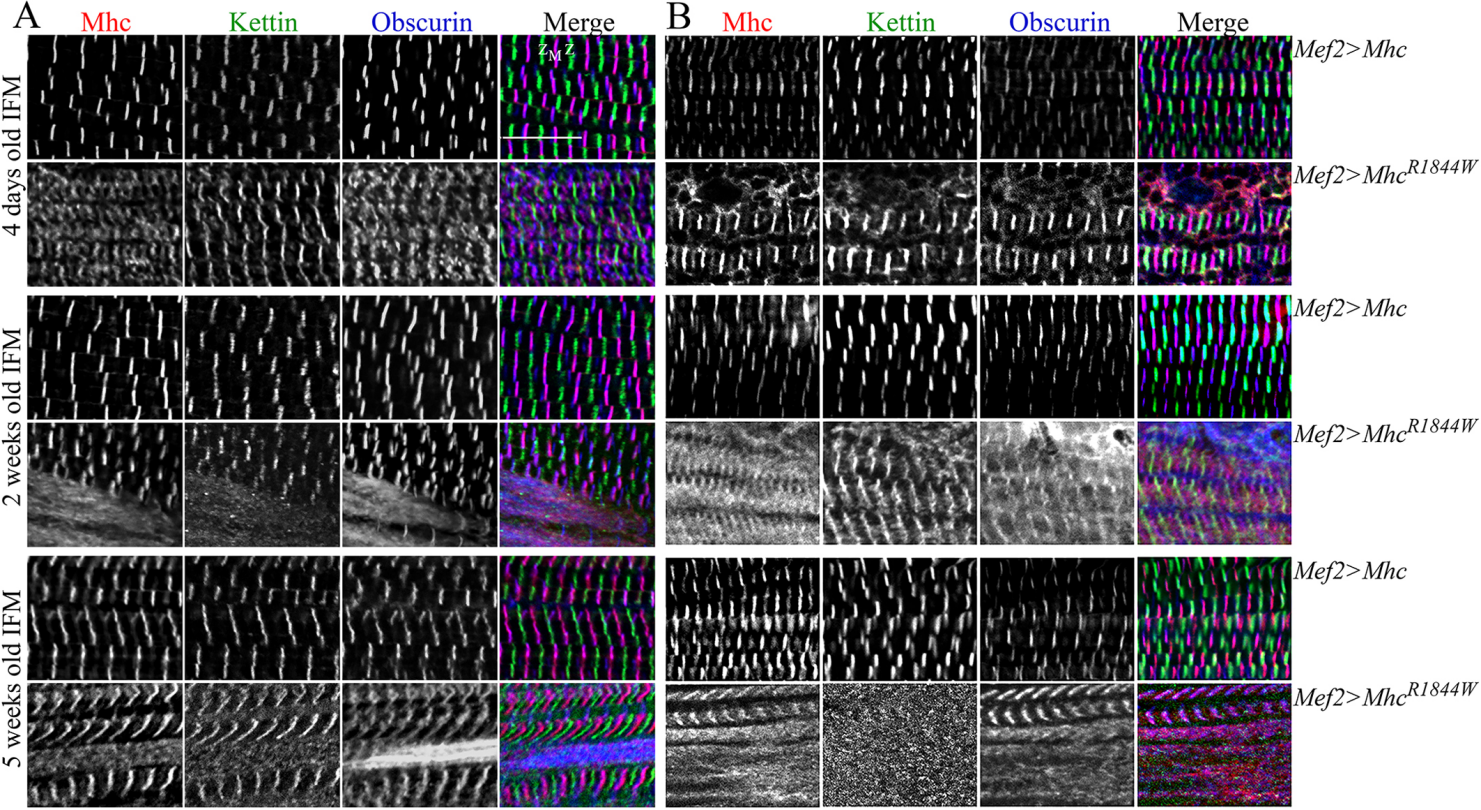
**Muscle morphology in IFMs of *eMhc^R1844W^* expressing adult flies.** (A,B) IFMs were labelled for Mhc, Kettin/Titin and Obscurin in 4-day-old, and 2- and 5-week-old adult flies kept at 25°C (A) and 29°C (B). Each panel shows *Mef2>eMhc* control animals (*w^−^*;*Mhc^1^,UAS-eMhc/+;Mef2-Gal4/^+^*) (top) and *Mef2>eMhc^R1844W^* animals (*w^−^*;*Mhc^1^,UAS-eMhc^R1844W^/^+^;Mef2-Gal4/^+^*) (bottom) for each age group*.* In both A and B, control flies showed parallel periodic striations across the IFMs. (A) *Mef2>eMhc^R1844W^* animals showed progressive disruptions in sarcomeric structure with age. Four-day-old *Mef2>eMhc^R1844W^* flies displayed less structured and defined A-bands and M-bands. Two- and 5-week-old adult myofibrils of *Mef2>eMhc^R1844W^* flies showed myofibril atrophy with undefined A-bands, M-bands and Z-disks. (B) At 29°C, IFMs of 4-day-old, and 2-week-old adult *Mef2>eMhc^R1844W^* flies, displayed severe sarcomeric disruption with few intact sarcomere structures. Five-week-old adult *Mef2>eMhc^R1844W^* flies were frequently missing defined sarcomere structures. Z and M indicate Z-disks and M-bands, respectively. Scale bar: 10 µm.

Different Mhc isoforms are normally expressed in larval and IFM of adult flies due to developmentally and spatially regulated alternative RNA splicing of a single *Drosophila* muscle *mhc* gene, (Bernstein et al., 1983). Therefore, to explore potential progressive effects of the *eMhc^R1844W^* allele on sarcomere structure, we analysed the IFMs of 4-day-old, and 2- and 5-week-old adult flies at 25 and 29°C. Immunofluorescence analyses of Mhc, Sallimus (Kettin/Titin) and Obscurin revealed a disrupted sarcomere structure with few intact sarcomeres in adult flies overexpressing *eMhc^R1844W^* compared to flies overexpressing *eMhc*. As we observed previously, at 25°C, IFMs of 4-day-old adult flies generally displayed less structured and separated Mhc-containing A-bands and M-bands, whereas Sallimus (Kettin/Titin) was detected in the distinct but slightly disorganised Z-disks (Fig. 4A, upper panel). Sarcomeric irregularities were more evident in 2- and 5-week-old animals, which exhibited some areas with undefined A-bands, M-bands and Z-disks in the IFM at 25°C. Furthermore, regions of myofibril atrophy could be seen in 5-week-old flies (Fig. 4A,B, lower panel). At 29°C, IFMs of 4-day-old, and 2- and 5-week-old adult flies, displayed severe sarcomeric disruption (Fig. 4B). Structured sarcomeric organisation was barely evident in 5-week-old flies at 29°C (Fig. 4B, lower panel). Thus, expression of *eMhc^R1844W^* appears to result in a progressive worsening of adult muscle phenotype in an expression level- and age-dependent manner that is not seen with eMhc.

### Ultrastructural impairment on expression of eMhc^R1844W^

**Fig. 5.**
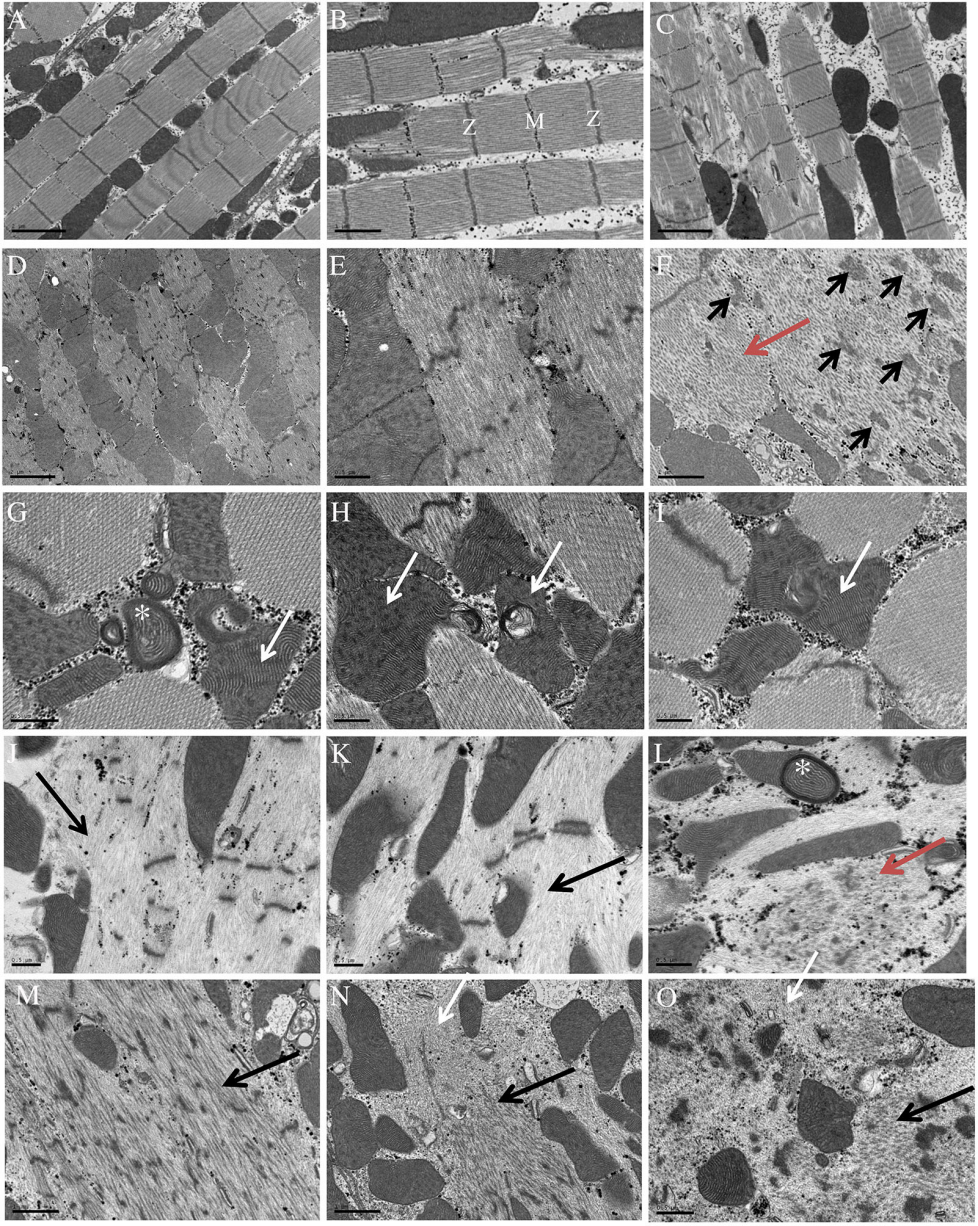
**Transmission electron micrographs of IFMs in *eMhc^R1844W^* expressing flies.** Transmission electron micrographs of IFMs in 7-day-old control (A-C), *Mef2>eMhc^R1844W^ (w^−^;Mhc^1^,UAS-eMhc^R1844W^/^+^;Mef2-Gal4/^+^)* (D-I)*, Mef2>eMhc^R1844W^/Mhc^10^* flies (*w^−^;Mhc^1^,UAS-eMhc^R1844W^/Mhc^10^;Mef2-Gal4/^+^*) (J-L), and *Mef2>eMhc/Mhc^10^* flies (*w^−^;Mhc^1^,UAS-eMhc/Mhc^10^;Mef2-Gal4/^+^*) (M-O), at 25°C. (A-C) Control flies showed well-preserved sarcomere structure with well-defined Z-disks, A-bands and M-bands. (D-F) Transverse sections of *Mef2>eMhc^R1844W^* flies revealed fibres with prominent abnormalities in the sarcomeric structure with lack of I-bands and M-bands and disrupted Z-disks. (E) A higher magnification of sarcomeric disorganization with absence of I-bands and M-bands and disrupted Z-disks in longitudinal sections. (F) Areas with thick filament accumulation (red arrow) and small aggregates of Z-disk-derived material (black arrows). (G-I) Apart from thick filaments accumulation, there were prominent mitochondria abnormalities with abnormal cristae (asterisk in G) and small electron-dense inclusions (white arrows in G-I). (J,K) IFMs of *Mef2>eMhc^R1844W^/Mhc^10^* flies revealed a severe loss of thick filament with selective A-band loss (black arrows) and preserved I-band and Z-disks. (L) Small areas containing disorganised thick filaments (red arrow) and abnormal mitochondria (asterisk). (M-O) IFMs of *Mef2>eMhc/Mhc^10^* flies revealed severe sarcomere disorganization with fragmented Z-disks. Thick filaments were preserved (black arrows, M-O), except in small areas that were devoid of thick filaments (white arrows, N and O). The sarcomeric structural parts, Z-disks and M-bands, are labelled. Scale bars: 2 µm (A,C,D); 1 µm (B,F,M,N); 0.5 µm (E,G-L,O).

Ultrastructural analysis of the IFM was performed on 7-day-old adult flies at 25°C. Well-organised sarcomeres with preserved Z-disk and M-bands were observed in IFMs of control flies (*Mef2>eMhc*) (Fig. 5A-C). Only small morphological abnormalities were identified in IFMs of flies with overexpression of *eMhc* (Fig. 5B,C). In contrast, in the IFMs of *Mef2>eMhc^R1844W^* flies, fibres displayed severe sarcomeric disorganization. Neither I-bands (the zone in which the thin filaments do not overlap the thick filaments) nor M-bands were visible and the Z-disks were disrupted (Fig. 5D,E, higher magnification). Thick filament accumulation (Fig. 5F, red arrow), small aggregates of electron-dense material of Z-disk origin (Fig. 5F, black arrows) and abnormal mitochondria (Fig. 5G-I) (asterisk in G and white arrows in H and I) were observed in the IFMs of *Mef2>eMhc^R1844W^* flies. More severe ultrastructural abnormalities, including extensive loss of thick filaments with selective A-band loss and preservation of I-band and Z-disks, were observed in the IFMs of *Mef2>eMhc^R1844W^/Mhc^10^* flies (Fig. 5J,K, black arrows). In addition, there were small collections of disorganised thick filaments (Fig. 5L, red arrow) and abnormal mitochondria (Fig. 5L, asterisk). In the IFMs of *Mef2>eMhc/Mhc^10^* flies, sarcomeres were disorganised and had severe Z-disk fragmentations (Fig. 5M-O). However, in contrast to the IFMs of *Mef2>eMhc^R1844W^/Mhc^10^* flies, which exhibited an almost complete absence of thick filaments, *Mef2>eMhc/Mhc^10^* flies had easily recognizable thick filaments. Only small occasional regions were devoid of myosin filaments (Fig. 5N,O, arrows).

### Overexpression of Abba suppresses eMhc^R1844W^- induced muscle defects

**Fig. 6.**
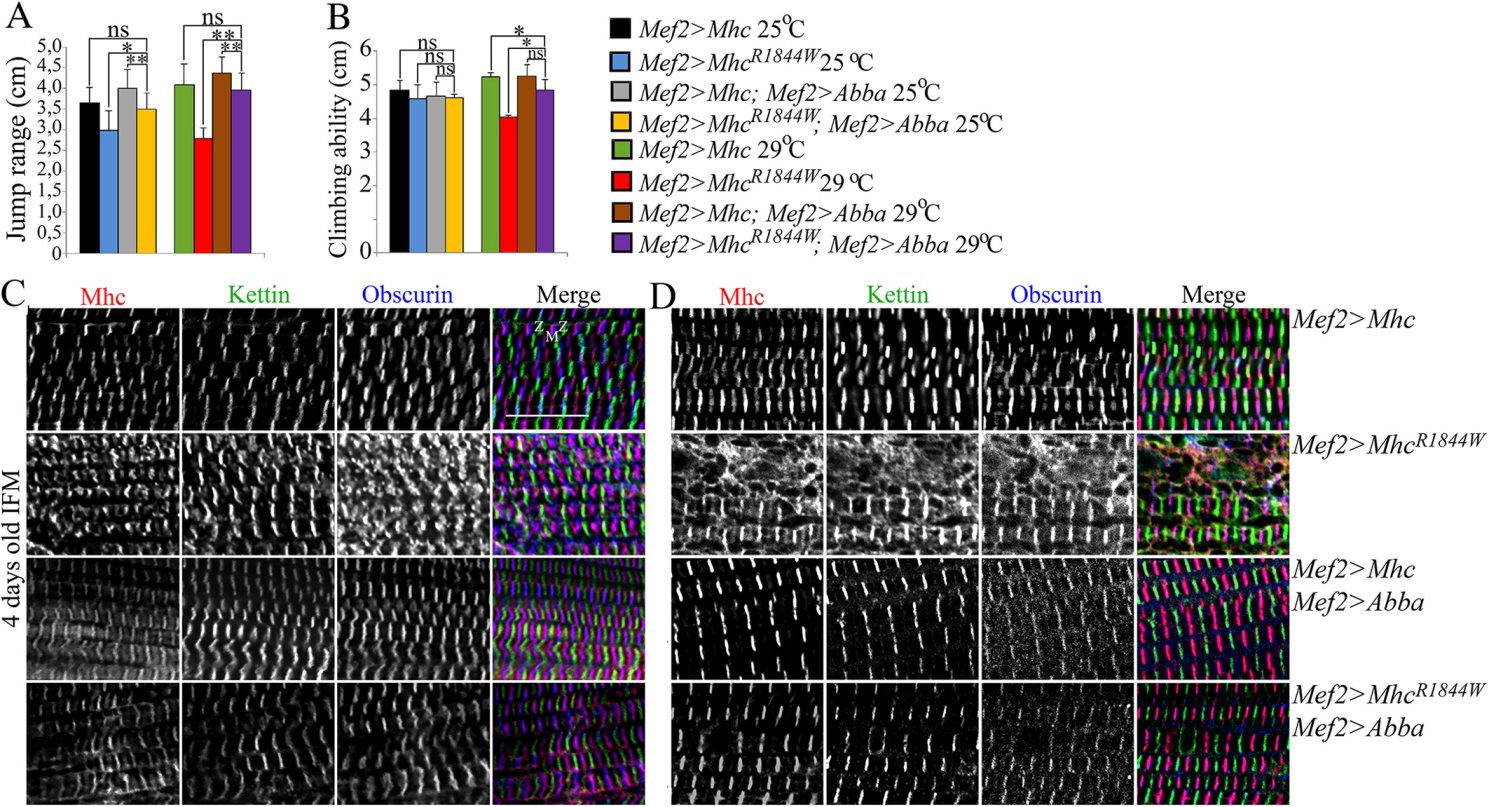
**Rescue of 4-day-old *Mef2>eMhc^R1844W^* fly muscle function and morphology with overexpression of Abba.** (A) Jump ability of *Mef2>eMhc^R1844W^* flies was restored upon overexpression of Abba (*Mef2>eMhc^R1844W^*; *Mef2>Abba*) to levels comparable with controls *Mef2>eMhc*, at both temperatures at 4 days of age. (B) Climbing ability of *Mef2>eMhc^R1844W^* and control animals was comparable at 25°C, and no changes were observed on overexpression of Abba. The climbing ability of *Mef2>eMhc^R1844W^; Mef2>Abba* was significantly improved upon overexpression of *Abba* at 29°C (*P*<0.05). (C-D) IFMs were labelled for Mhc, Kettin/Titin and Obscurin in 4-day-old adult flies at 25°C (C) and 29°C (D). (C) *Mef2>Mhc* control flies, with overexpression of Abba (*Mef2>eMhc; Mef2>Abba*), showed parallel periodic sarcomeric striations in the IFMs. Less defined sarcomeric structure was observed in the IFMs of *Mef2>Mhc^R1844W^* flies. *Mef2>eMhc^R1844W^* flies with overexpression of Abba (*Mef2>eMhc^R1844W^; Mef2>Abba*) at 25°C displayed almost structured periodic striations with slightly sarcomeric disorganisation. (D) Few intact sarcomeres were observed in the IFMs of *Mef2>Mhc^R1844W^* flies at 29°C. Overexpression of Abba in *Mef2>eMhc^R1844W^* flies (*Mef2>eMhc^R1844W^; Mef2>Abba*) at 29°C resulted in structured periodic sarcomeric striations, comparable with *Mef2>eMhc* control flies. The sarcomeric structural parts, Z-disks and M-bands, are labelled. Scale bar: 10 µm.

We have previously shown that Abba expression alleviates muscle defects associated with the *Mhc^K1728del^* mutation in a *Drosophila* model of Laing distal myopathy (Dahl-Halvarsson et al., 2018). Muscle defects induced by *Mhc^K1728del^* were completely suppressed by overexpression of *Abba* (Dahl-Halvarsson et al., 2018). In *Drosophila*, Abba (another B-box affiliate) belongs to the TRIM/RBCC protein family and is required for sarcomeric integrity during muscle formation and function, and *Abba* mutants display an abnormal accumulation of Mhc structures (Domsch et al., 2013; LaBeau-DiMenna et al., 2012). In humans, muscle-specific RING finger (MuRFs) proteins are members of the TRIM/RBCC superfamily of E3-ligases and regulate degradation of MyHCs. MuRF1 (also known as TRIM63) deficiency combined with a deleterious MuRF3 (also known as TRIM54) mutation leads to subsarcolemmal accumulation of myosin (Olive et al., 2015), similar to the muscle morphological features of MSM (Tajsharghi and Oldfors, 2013). We therefore investigated whether elevated levels of Abba expression have any effect on muscle defects induced by expression of *eMhc^R1844W^*. In flies overexpressing *eMhc^R1844W^*, overexpression of Abba did not result in a significant rescue of flight ability. However, overexpression of *Abba* was able to improve some muscle functions in *Mef2>eMhc^R1844W^* animals. The jump ability of mutant *Mef2>eMhc^R1844W^* flies was restored almost to that of the wild type upon muscle-specific expression of Abba at 25°C and 29°C (Fig. 6A). At 25°C, there was no difference in climbing ability between *Mef2>eMhc^R1844W^* and *Mef2>eMhc* control animals, and expression of Abba revealed no changes, but climb ability of mutant *Mef2>eMhc^R1844W^* flies was significantly improved by increased levels of Abba at 29°C (Fig. 6B). Thus, some muscle dysfunction caused by *eMhc^R1844W^* can be alleviated by increased levels of Abba, although flies remained flightless. To assess the morphology of the IFMs in *Mef2>eMhc^R1844W^* and *Mef2>eMhc^R1844W^;Mef2>Abba* animals, we again used antibodies against Mhc, Sallimus (Kettin/Titin) and Obscurin. Immunofluorescence analyses revealed a slightly sarcomeric disorganisation in *eMhc^R1844W^* flies with limited *Abba* overexpression at 25°C (Fig. 6C). In contrast, an apparent improvement of sarcomeric organization was revealed in these flies with distinct Z-disks, M-bands and Mhc-containing thick filaments at 29°C (Fig. 6D). A nearly complete rescue at the morphological level was observed in the entire muscle in repeated experiments. Moreover, the distinct areas of increased myosin immunofluorescence that were observed in 4-day-old *Mef2>eMhc^R1844W^* flies were not detected in *Mef2>eMhc^R1844W^* flies that overexpressed *Abba* at 29°C (Fig. 6D). These findings indicate that elevated levels of *Abba* are able to relieve muscle morphological defects associated with the e*Mhc^R1844W^* allele in the presence of wild-type *Mhc*.

**Fig. 7.**
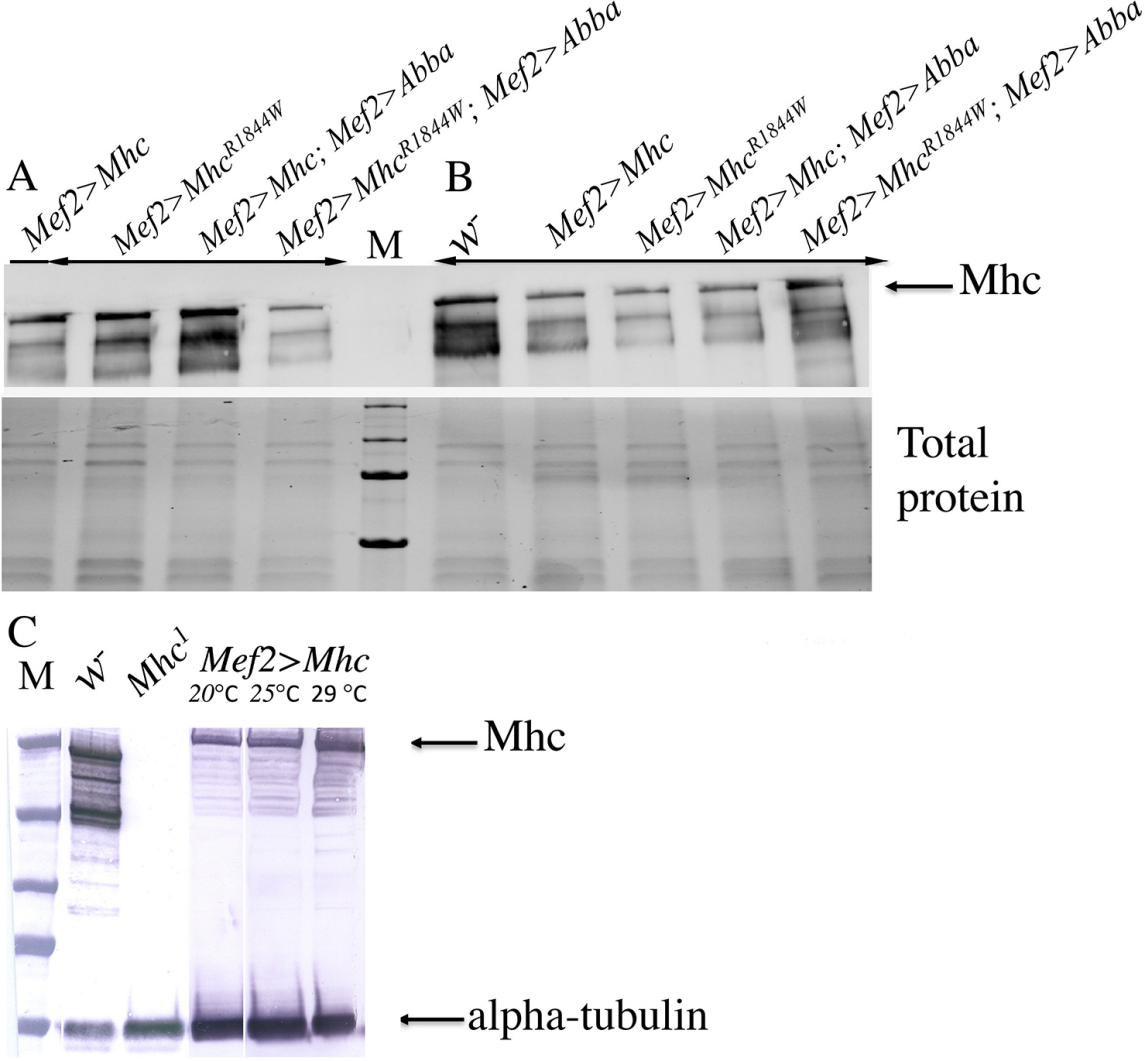
**Mhc expression levels in transgenic lines.** (A,B) Relative Mhc expression levels in 4-day-old transgenic lines were determined using immunoblot analysis. Protein extracted from transgenic lines carrying UAS-*eMhc* (*Mef2>eMhc*) or UAS-*eMhc^R1844W^* (*Mef2>eMhc^R1844W^*), and transgenic flies used in the assessment of rescuing the effect of simultaneous overexpression of *eMhc* and Abba (*Mef2>eMhc; Mef2>Abba*) or UAS-*eMhc^R1844W^* (*Mef2>eMhc^R1844W^; Mef2>Abba*), at 25°C and 29°C, were analysed. *w^−^* strain flies were used as controls. Similar expression levels of eMhc in animals with overexpression of e*Mhc^R1844W^*, or e*Mhc* allele with or without overexpression of *Abba* at 25°C (A) or 29°C (B), are indicated. Myosin and total protein expression are indicated (arrow). (C) Assessment of the overexpression ability of *Mef2-Gal4* system. The *Mef2-Gal4* driver induced the expression of *UAS-eMhc* in the *Mhc^1^* null strain, when compared to *Mhc^1^* null embryos. Lower expression levels of e*Mhc* allele in *Mhc^1^* embryos at 25°C or 29°C, compared with myosin levels in *w^−^* wild-type control, are indicated. Myosin and alpha-tubulin expressions are indicated (arrows).

As the simultaneous overexpression of both e*Mhc* and *Abba* is under the control of the *Mef2* promoter and the GAL4 system driver, the expression level of eMhc in the transgenic lines was determined. Results obtained from the immunoblot assay indicated similar expression levels of eMhc in animals with overexpression of e*Mhc^R1844W^* or the e*Mhc* allele with or without overexpression of *Abba* at 25°C or 29°C (Fig. 7A,B). Furthermore, results from the immunoblot assay confirmed that the expression level of the *UAS-eMhc* in *Mhc^1^* embryos induced by the *Mef2-Gal4* driver was clearly lower than the expression level of endogenous myosin in *w^−^* control flies at 25°C or 29°C (Fig. 7C). These results ruled out the possibility of extreme expression levels of myosin under the control of the *Mef2* promoter, which might titrate out other sarcomeric components and consequently lead to impaired muscle morphology. Furthermore, in flies overexpressing *eMhc^R1844W^*, Abba overexpression alleviates the pathological phenotypes of muscle structure, suggesting that the disruptions of the sarcomeres observed in the fly muscle were associated with the mutant allele rather than a high level of myosin protein.

Collectively, these data show that Abba expression levels modulate the muscle phenotype observed in *Mef2>eMhc^R1844W^* animals, with elevated Abba levels restoring muscle morphology. Although increased Abba levels enhanced some muscle function, flight ability in flies was not rescued, suggesting that embryonic Mhc is dominant antimorphic.

## DISCUSSION

Several *in vitro* and *in vivo* studies have been performed to investigate molecular mechanisms involved in the pathogenesis of diseases caused by mutations in the rod region of muscle MyHC. Biochemical and biophysical characterization of the effects of the myosin storage myopathy mutations in the LMM region have suggested adverse effects of the mutations in the ability of the protein to form stable and functional thick filaments (Armel and Leinwand, 2009), and that each mutation has a unique effect on the ability to form accumulated materials (Dahl-Halvarsson et al., 2017). However, it is still unclear why Laing distal myopathy and MSM are associated with different muscle pathologies.

MSM models of 1-day-old flies were previously generated to study the impact of three MSM mutations, including the R1845W mutation, on muscle performance (Viswanathan et al., 2017). Flies with exclusive expression of mutant *Mhc^R1844W^* allele in IFM exhibited severe impairment of flight and jump ability concomitant with progressive disrupted muscle ultrastructure and myosin aggregates (Viswanathan et al., 2017). In this study, we have established an MSM model of the recurrent R1845W mutation in *Drosophila* to provide insight into the mechanisms governing the muscle involvement and the development of the disease in an extended age- and dose-dependent manner. Furthermore, we explored a potential therapeutic approach involving myosin degradation via the UPS in MSM. The *Mhc^R1844W^* allele, corresponding to the recurrent R1845W mutation in slow/β cardiac MyHC, represents a disease model for the first and the most common MSM-causing mutation (Tajsharghi et al., 2003; Tajsharghi et al., 2007; Tajsharghi and Oldfors, 2013). The disease is slowly progressive, the severity of myopathy is not related to age, and evidence of cardiomyopathy associated with the myopathy is usually not found (Tajsharghi et al., 2003; Tajsharghi and Oldfors, 2013). Muscle biopsy is characterised by stored material with intense immunoreactivity to slow/β cardiac MyHC (Tajsharghi and Oldfors, 2013). The inclusion bodies are not limited by a membrane but the stored material can be seen between partly disintegrated myofibrils in the vicinity of the main storage body, and at higher magnification they have a granular appearance (Tajsharghi and Oldfors, 2013).

Our analyses of *eMhc^R1844W^ Drosophila* muscle revealed some large areas with increased myosin immunofluorescence, indicating myosin accumulation, reminiscent of the stored material observed in muscle biopsies from patients carrying MSM-associated mutations, including the R1845W mutation. A near total absence of structured sarcomeric organisation was evident in 5-week-old mutant flies with high expression level of *Mhc^R1844W^* allele. Thus, *eMhc^R1844W^* appears to result in an age- and expression-level-dependent progressive worsening of adult muscle phenotype. In addition, it is likely that the mutant myosin is more subject to denaturation at an elevated temperature correlated with a higher expression level, leading to enhanced phenotypes. The expression of the mutated *eMhc^R1844W^* allele in an otherwise wild-type situation caused sarcomere disruption, indicating that expression of the mutant allele is the underlying cause of the phenotype. This correlated with ultrastructural findings demonstrating sarcomeric disorganization and areas with accumulation of thick filaments, which failed to integrate into sarcomeres resembling the situation observed in the muscle biopsies of MSM patients (Tajsharghi and Oldfors, 2013). However, unlike muscle biopsies of MSM patients, the inclusions did not appear as granular material. In addition, the Z-disks appeared disrupted. Together, this indicates the actual impact of the R1844W mutation on muscle architecture, with decreased myosin filament stability and myofibrillar disassembly leading to a progressive myofibril degeneration. This muscle pathology differs from the abnormalities observed in a *Drosophila* model of the Laing distal myopathy (*Mhc^K1728del^* flies), which shows large areas of sarcomeric disorganization, with Z-disk streaming and extensions as the main muscle morphological features (Dahl-Halvarsson et al., 2018).

Consistent with their aberrant muscle architecture, *eMhc^R1844W^* flies also display defects in flight, jumping and climbing ability, as well as a reduced lifespan. Impaired larval muscle functions, such as turning from belly-up to belly-down, were observed in *eMhc^R1844W^* mutants during first and second larval instar, and the severity of muscle weakness is related to the expression level of the *eMhc^R1844W^* allele. The phenotype is consistent with the demonstrated severe sarcomere abnormalities, disintegration of sarcomeric structure and mislocalization of core components, such as UNC45, Kettin/Titin and M-bands, indicating a marked impact of the mutated myosin on muscle function.

IFMs of *Mef2>eMhc^R1844W^/Mhc^10^* animals, with exclusive expression of the *Mhc^R1844W^* allele, revealed more severe ultrastructural abnormalities, with completely disrupted myofibrillar structures, very few intact sarcomeres and a massive loss of thick filaments with selective A-band loss and preservation of I-band and Z-disks. These ultrastructural features are identical to those observed in muscle biopsies from patients with critical illness myopathy (Larsson et al., 2000), which results from massive degradation of MyHC by the UPS. Interestingly, MuRF1 has been found to be upregulated in skeletal muscle from critical illness myopathy patients, demonstrating the involvement of MuRF1 in thick filament degradation (Larsson et al., 2000).

The development of cardiomyopathy and skeletal myopathy associated with subsarcolemmal myosin accumulation and non-ubiquinated aggregates of fragmented sarcomeres were observed in a patient with compound MuRF1 (TRIM63) deficiency and a deleterious MuRF3 (TRIM54) missense mutation (Olive et al., 2015). Highly similar features were previously observed in mice deficient for MuRF1 and MuRF3 (Fielitz et al., 2007). The TRIM/RBCC family includes the MuRF E3 ubiquitin ligases, which catalyse the degradation of MyHCs via the UPS in the muscle (Freemont, 2000; Glass and Roubenoff, 2010). In *Drosophila*, Abba protein, which is homologous to human TRIM E3 ubiquitin ligase, has a fundamental role in maintaining myofibril bundling and sarcomeric integrity during muscle development and usage (Domsch et al., 2013; LaBeau-DiMenna et al., 2012). Flies with Abba deficiency displayed similar muscle phenotypes, as seen in double knockout mice and in patients with compound MuRF1 deficiency and a deleterious MuRF3 mutation (Domsch et al., 2013; LaBeau-DiMenna et al., 2012).

A lack of ubiquitin in the inclusions found in the patient with compound MuRF1 deficiency and a deleterious MuRF3 missense mutation (Olive et al., 2015), and in MSM patients (Tajsharghi et al., 2003; Tajsharghi and Oldfors, 2013), as well as in the IFMs of flies with MSM-associated mutations (Viswanathan et al., 2017), indicates that mutant myosin in the aggregates are not targeted for degradation by the UPS. Thus, thick filament protein aggregations that contribute to the disease phenotype can be caused by MSM-associated mutations in *MYH7* and defective degradation of myosin by the UPS in muscle. This offers an efficient tool for exploring potential therapeutic approaches involving UPS to alleviate thick filament protein aggregation and being beneficial in MSM.

Recently, we showed that overexpression of *Abba* can restore muscle function and morphology in a *Drosophila* model of Laing distal myopathy with *Mhc^K1728del^* mutation. Abba overexpression fully suppressed the phenotypic effects of heterozygous *Mhc^K1728del^*. However, reduced Abba levels in animals heterozygous for *Abba^MJO0348^* enhanced the muscle defects associated with the heterozygous *Mhc^K1728del^* condition, showing that Abba functions to counteract the deleterious effects of *Mhc^K1728del^* (Dahl-Halvarsson et al., 2018). Here, we further show that overexpression of *Abba* can restore muscle morphology in flies with *eMhc^R1844W^* mutation. Although some muscle dysfunction was enhanced, lack of flight ability was, however, not rescued by overexpression of *Abba* in flies with *eMhc^R1844W^* mutation. This can be explained by a dominant antimorphic feature of embryonic Mhc, as previously demonstrated (Wells et al., 1996). In addition, the results from immunoblot analyses indicated similar expression levels of eMhc in animals with overexpression of e*Mhc^R1844W^* or e*Mhc* allele, with or without overexpression of *Abba*. This ruled out the possibility that an insufficient amount of Abba due to competitive expression levels between *Abba* and *eMhc* or *eMhc^R1844W^*, given that the muscle-specific *Mef2-Gal4* driver concurrently induced the expression of both *UAS-Abba* and *UAS-eMhc* or *UAS-eMhc^R1844W^*, was unable to adequately rescue the flight muscle function. Partial rescue of muscle function in *eMhc^R1844W^* flies may also reflect the more severe impact of the R1844W mutation compared to the Laing models, in which we observed complete rescue of muscle phenotypes in the *Mhc^K1728del^* flies with overexpression of Abba (Dahl-Halvarsson et al., 2018). This is in line with the failure to generate an MSM model in *Drosophila* by CRISPR/Cas9-mediated genome editing. Given that we were able to modify the R1844 site without creating an amino acid change, it is possible that the mutant *Mhc^R1844W^* allele resulted in lethality and thus we were unable to identify and generate a fly line harbouring the *Mhc^R1844W^* allele. The milder effect of the *MYH7 R1845W* mutation in patients and on embryonic viability might be explained by the existence of embryonic and perinatal MyHC isoforms, encoded by *MYH3* and *MYH8*, respectively, which are expressed during fetal development and muscle generation (Feghali and Leinwand, 1989; Karsch-Mizrachi et al., 1989).

Taken together, data from the current study indicates that the observed variation in clinical and pathologic phenotypes of slow/β cardiac MyHC mutation is mutation-specific and that it may in part be due to the genetic background related to the existence of E3-ligase modifier genes that may reduce or enhance the impact of the mutation, as previously suggested in our study of a Laing distal myopathy model in *Drosophila* (Dahl-Halvarsson et al., 2018).

In conclusion, we have generated a *Drosophila* MSM model, and show that severity of the disease correlates with the expression levels of the mutated allele. In addition, the muscle morphology phenotypes and some muscle dysfunctions associated with the *Mhc^R1844W^* condition are suppressed by overexpression of the TRIM family protein Abba.

## MATERIALS AND METHODS

### Experimental approaches to generate a Mhc^R1844W^ mutant allele

#### CRISPR/Cas9 genome editing

The engineering of the *Drosophila* genome was performed via CRISPR/Cas9-mediated genome editing. The targeting sequence (5′-GCTGAGCTTCCAGTCCGAGGAGG-3′) was cloned into the pCFD3-dU6:3gRNA plasmid and co-injected with a 177 bp long single-stranded oligodeoxynucleotide (ssODN) in fly embryos expressing Cas9 during oogenesis (*y^1^ M{vas-Cas9}ZH2A w^1118^*) (BestGene). The ssODN donor encompassed the wild-type R1844 or R1844W mutation (CGT>TGG) and a silent mutation located at position 5687 (NM_165191) (GGACC>AGACC) to remove an AvaII enzyme digestion site in the presence of the R1844W mutation. The genomic region of interest was amplified by PCR from whole-fly extracts and digested with AvaII to identify the R1844W mutation. The result was confirmed by sequencing of exons over the entire *Mhc* locus. This may indicate a major impact of the heterozygous *Mhc^R1844W^* allele on viability. The heterozygous wild-type *Mhc* allele was maintained as a stock over a *CyO* balancer chromosome carrying *Deformed>YFP*. Heterozygous *Mhc^R1844W^* flies could never be maintained despite repeated injections.

### Construction of UAS-Mhc

The pUASTaAttB vector (FlyBase ID: FBmc0003002) was used as the construct backbone. Available AttB sites flanking the upstream activation sequence (UAS) promoter and cDNA fragment allow site-directed insertion into the *Drosophila* genome upon injection into a specific strain that carries corresponding AttP-sites at a given genomic location. Two plasmids covering the embryonic *Mhc* (*eMhc*) cDNA, a 5′ part (covering exons 1-12) and a 3′ part (covering exons 12-19) both carried in the BlueScript-KS vector, were kindly provided by S.I. Bernstein (San Diego State University, CA, USA). The two *eMhc* cDNA fragments were combined by excision of the 5′ part with XbaI and ApaI, followed by subcloning into the BlueScript-KS vector containing the 3′ fragment cut with the same restriction enzymes. The resulting full- length *eMhc* cDNA was excised using NotI and KpnI restriction enzymes and ligated into the pUASTaAttB vector, which was digested with the same enzymes. The *eMhc* R1844W mutation (corresponding to R1845W in humans) was introduced by QuikChange II (Agilent Technologies) into the full-length wild-type *eMhc* cDNA fragment (GenScript). The wild-type and mutated *eMhc* constructs were validated by sequencing of the entire cDNAs.

### *Drosophila* genetics and lines

Fly culture, crosses and analyses were performed on standard fly food and at room temperature (22°C), unless otherwise stated. The *w^1118^* (*w^−^*) line was used as a wild-type genetic background. Transgenic lines carrying *UAS-eMhc* or *UAS-eMhc^R1844W^* on a attP landing site on the second chromosome were generated in a *w^−^* background (*w^−^*;*UAS-eMhc* and *w^−^*;*UAS-eMhc^R1844W^*) (BestGene) and balanced over *CyO*, *Deformed*-*YFP* (Bloomington Stock Center). Flies carrying the loss-of-function allele *Mhc^1^* or the amorphic *Mhc^10^* allele that results in undetectable levels of Mhc in the IFM and jump muscle (Collier et al., 1990) were kindly provided by S.I. Bernstein (San Diego State University). Flies carrying *UAS-Abba* on the third chromosome (*w^−^; UAS-Abba*) were kindly provided by Dr H. Nguyen (University of Erlangen-Nuremberg, Germany). The muscle-specific *Mef2-Gal4* driver (expressing Gal4 in muscle lineages), obtained from the Bloomington Stock Center (stock number 27390), was used to induce *UAS-eMhc* or *UAS-eMhc^R1844W^* overexpression. As *Gal4* activity increases with temperature, the level of transgene expression can be modulated by altering the rearing temperature. This was used to correlate expression levels with phenotype.

The phenotypic effect of the *Mhc^R1844W^* allele was studied by overexpression of either mutated or wild-type *eMhc* in flies with a heterozygote null *Mhc* allele (*Mhc^1^*) background (*w^−^*;*Mhc^1^,UAS-eMhc^R1844W^/^+^;Mef2-Gal4/^+^* or *w^−^*;*Mhc^1^,UAS-eMhc/^+^;Mef2-Gal4/^+^*) at 25°C and 29°C. The exclusive effect of the *Mhc^R1844W^* allele in flight and jump muscles was further studied by overexpression of mutated or wild-type *eMhc* in flies carrying the amorphic *Mhc^10^* allele (*w^−^*;*Mhc^1^,UAS-eMhc^R1844W^/Mhc^10^;Mef2-Gal4/^+^* and *w^−^*;*Mhc^1^,UAS-eMhc/Mhc^10^;Mef2-Gal4/^+^*), at 25°C and 29°C. The rescuing effect of overexpression of *Abba* in animals expressing *eMhc^R1844W^* (*w^−^;Mhc^1^,UAS-eMhc^R1844W^/^+^*;*UAS-Abba*/*Mef2-Gal4* or *w^−^;Mhc^1^,UAS-eMhc/^+^;UAS-Abba/Mef2-Gal4*) were analysed at 25°C and 29°C.

### Larval motility assays

The crawling assay was performed with third instar larvae, as described previously (Nichols et al., 2012; Dahl-Halvarsson et al., 2018). Briefly, single larvae were transferred to 9 cm apple juice plates and the position of the animal was recorded for 1 min to trace the movements. At least twenty animals of each genotype were analysed. In the larval turning assay (Estes et al., 2011; Dahl-Halvarsson et al., 2018), third instar larvae were placed on apple juice plates and gently rolled ventral side up. The time taken for larvae to return to a dorsal side up position and continue their forward movement was recorded.

### Jumping and flight assays in adult flies

Four-day-old, 2-week-old and 5-week-old flies were evaluated for jump and flight abilities. The jump assay was essentially performed as described previously (Swank et al., 2002) (Dahl-Halvarsson et al., 2018); jump ability was defined as the horizontal distance a fly was able to jump from a 7-cm-high platform. Wings were removed before jump testing. Twenty flies per genotype were assessed in ten replicates. Flight ability was assessed for 20 min at room temperature in a transparent vial (9 cm high, internal diameter 2.6 cm) with a light source at the top of the vial to encourage flies to fly. The vial was agitated every 5 min. Flies that were not able to perform flight motion within 20 min, and fell straight to the bottom when the bottle was tapped, were considered flightless. The experiment was recorded and individual flies were carefully analysed. Twenty flies per genotype were assessed in five replicates.

### RING assay

Four-day-old, 2-week-old and 5-week-old flies were evaluated for negative geotaxis, an innate escape response driven by mechanical stimulation of the flies. The RING assay was performed as described previously (Dahl-Halvarsson et al., 2018). Briefly, the position of the flies in the tubes were captured in digital images taken 3 s after initiating the behaviour/tapping. The RING was assessed in a total of five consecutive trials separated by 1 min of rest for each genotype and age group at room temperature in a transparent vial (9 cm high, internal diameter 2.6 cm). After completed trials, captured images were used to score the height climbed for each fly. Climbing ability was calculated as the average of five sequential trials. Ten flies per genotype were assessed in five replicates.

### Viability and lifespan of adult flies

Animals were kept at 25°C or 29°C until they were eclosed. Ten flies of the same sex were then kept in 12 ml vials with standard fly food at room temperature (22°C). Flies were regularly transferred to new food vials, and dead flies were recorded every three days until day 30 (0, 3, 6, 9, 12, 15, 18, 21, 24, 27 and 30). Day 0 denotes eclosion. Fly lifespan analysis was performed in three replicates.

### Immunofluorescence and confocal microscopy of larval and adult flies

The immunofluorescence analysis was performed as described previously (Dahl-Halvarsson et al., 2018). Dissected body wall muscles from third instar larvae and IFM from pupae and adult flies (4-day-old and 2- and 5-week-old flies) were fixed with 4% formaldehyde for 10 min. Samples were permeabilised in PBS containing 2% bovine serum albumin and 1% Triton X-100 for 30 min, and then incubated overnight at 4°C with primary antibodies. The following primary antibodies were used: IgG mouse anti-Myosin (1:100, kindly provided by Dr from J.D. Saide, Boston University, MA, USA), IgG1 rat anti-Titin/Kettin MAC155 (1:100, Abcam, ab50585), IgG rabbit anti-Obscurin (1:100, kindly provided by Dr B. Bullard, University of York, UK) and rabbit anti-Unc45 (1:500 kindly provided by S.I. Bernstein). The following secondary antibodies, all obtained from Jackson ImmunoResearch, were used: goat IgG anti-Mouse Cy3 (115-165-146, 1:1000), donkey IgG anti-rabbit Alexa Fluor 647 (711-605-152, 1:600) and donkey IgG ant-rat Alexa Fluor 488 (712-545-150, 1:200). Samples were mounted in Fluoromount-G and imaged using a Confocal LSM800 or LSM700 microscope (Zeiss) with a 63× objective. Images were processed using Photoshop (Adobe).

### Ultrastructural analysis

Preparation of samples for transmission electron microscopy of IFMs in adult flies was carried out as described previously (Burkart et al., 2007; Dahl-Halvarsson et al., 2018). Samples were viewed on a JEOL 1011 electron microscope and images were captured using a Gatan 782 camera.

### Protein analysis by Western blot

Relative Mhc expression levels in transgenic lines were determined using immunoblot analysis. Transgenic lines carrying *UAS-eMhc* (*w^−^*;*Mhc^1^,UAS-eMhc/^+^;Mef2-Gal4/^+^*) or *UAS-eMhc^R1844W^* (*w^−^*;*Mhc^1^,UAS-eMhc^R1844W^/^+^;Mef2-Gal4/^+^*) at 25°C and 29°C, and transgenic flies used in the assessment of rescuing the effect of overexpression of *Abba* expressing *eMhc* (*w^−^;Mhc^1^,UAS-eMhc/+;UAS-Abba/Mef2-Gal4*) or *eMhc^R1844W^* (*w^−^;Mhc^1^,UAS-eMhc^R1844W^/^+^*;*UAS-Abba*/*Mef2-Gal4*) at 25°C and 29°C, were used. *w^−^* and null *Mhc* allele (*Mhc^1^*) strain flies were used as controls. Protein was extracted from *Mhc^1^* and *UAS-eMhc* embryos, and 4-day-old flies from each line homogenised in radioimmunoprecipitation assay buffer (Thermo Fisher Scientific, 89901) on ice. Western blot analysis was performed using a Mini-PROTEAN TGX 8-20% gradient gel (Bio-Rad). Protein was blotted onto LF-PVDF membrane (8 min, 25 V and 2.5A) using a Trans-Blot Turbo Transfer System (Bio-Rad). Blots were subsequently blocked for 1 h in 5% milk in TBST buffer (0.1% Tween 20 and 150 mM NaCl in 10 mM Tris–HCL, pH 7.4) as per the manufacturer's recommendations. Blots were probed overnight at 4 degrees with antibodies diluted in PBST (0.1% Tween 20 in PBS). The primary antibodies used were *Drosophila* myosin (3E8-3D3), developed by Boston University, and alpha-Tubulin (12G10), developed by The University of Iowa. Both antibodies were used at 0.2 μg/ml and obtained from the Developmental Studies Hybridoma Bank, created by the Eunice Kennedy Shriver National Institute of Child Health and Human Development (National Institutes of Health) and maintained at The University of Iowa. The Secondary antibodies used for detection were: starbright goat anti-mouse IgG (1:2500, 12004158, Bio-Rad). All wash stages were 3×10 min in TBST 0.1%. Secondary antibodies were incubated for 1 h at room temperature.

### Statistical analysis

Graphs and statistical comparisons were generated using IBM SPSS 20 Statistics. Statistical data are presented as mean±s.e.m. Survival data were analysed with the Kaplan–Meier estimator, and statistical comparisons were made with log-rank pairwise analysis. Statistical significance for locomotor effects was determined by an unpaired two-tailed Student's *t*-test. The mean difference was considered to be statistically significant at the 95% confidence level. Results were considered as not significant (ns) when *P*>0.05, very significant when *P*=0.01<*P*<0.05 (*) and extremely significant when *P*<0.001 (**). Figures were assembled with Adobe Photoshop and Illustrator CC 2015.5 (Adobe).
